# Support for and aspects of use of educational games in family medicine and internal medicine residency programs in the US: a survey

**DOI:** 10.1186/1472-6920-10-26

**Published:** 2010-03-25

**Authors:** Elie A Akl, Sameer Gunukula, Reem Mustafa, Mark C Wilson, Andrew Symons, Amir Moheet, Holger J Schünemann

**Affiliations:** 1 Department of Medicine, State University of New York at Buffalo, Buffalo, NY, USA; 2 Department of Family Medicine, State University of New York at Buffalo, Buffalo, NY, USA; 3 Department of Clinical Epidemiology & Biostatistics, McMaster University, Hamilton, ON, Canada; 4 Department of Internal medicine, University of Iowa, Iowa City, IA, USA; 5 Department of Medicine, University of Rochester, Rochester, NY, USA

## Abstract

**Background:**

The evidence supporting the effectiveness of educational games in graduate medical education is limited. Anecdotal reports suggest their popularity in that setting. The objective of this study was to explore the support for and the different aspects of use of educational games in family medicine and internal medicine residency programs in the United States.

**Methods:**

We conducted a survey of family medicine and internal medicine residency program directors in the United States. The questionnaire asked the program directors whether they supported the use of educational games, their actual use of games, and the type of games being used and the purpose of that use.

**Results:**

Of 434 responding program directors (52% response rate), 92% were in support of the use of games as an educational strategy, and 80% reported already using them in their programs. Jeopardy like games were the most frequently used games (78%). The use of games was equally popular in family medicine and internal medicine residency programs and popularity was inversely associated with more than 75% of residents in the program being International Medical Graduates. The percentage of program directors who reported using educational games as teaching tools, review tools, and evaluation tools were 62%, 47%, and 4% respectively.

**Conclusions:**

Given a widespread use of educational games in the training of medical residents, in spite of limited evidence for efficacy, further evaluation of the best approaches to education games should be explored.

## Background

In their efforts to comply with ACGME regulations, program directors are constantly on the lookout for innovative educational strategies [1]. One of the strategies that appear to be increasing in popularity is the use of educational games. In these games the learner "engages in some activity, looks back at the activity critically, abstracts some useful insight from the analysis and puts the results to work [2]." Educational games differ from other educational strategies in their use of prescribed settings constrained by rules, procedures, and their competitive nature [3].

The evidence supporting the effectiveness of educational games in graduate medical education is limited. A systematic review of the effects of educational games in health professionals could neither confirm nor refute their utility [4]. One systematic review found limited evidence that educational games could help mental health students improve their knowledge [5]. Another systematic review concluded that the available evidence did not confirm nor refute the utility of educational games as an effective teaching strategy for medical students [6].

Medical educators have used educational games to teach medical students [7] as well as medical residents [8]. Residency training programs have reported using these games for teaching [9], review [10] and evaluation purposes [11]. The topics covered in these games range from basic sciences [12] to clinical practice guidelines [8,13].

In the context of limited supporting evidence, it is valuable for both medical educators and medical education researchers to know the extent of use and support for this type of educational strategies. Indeed, a high level of use of educational games in spite of the limited current evidence for their efficacy will highlight the need for rigorous development and evaluation. At the same time, high levels of support increase the likelihood of adoption of this educational strategy when rigorously developed and tested tools become available. Primary care residency training programs in the United States appear to have interest in educational games and at the same time provide an opportunity for a widespread application of this educational strategy if proven effective. Thus, the objective of this study was to explore the support for and the different aspects of use of educational games in family medicine and internal medicine residency programs in the United States (US).

## Methods

### Study population

The study population consisted of the program directors of family medicine and internal medicine residency programs in the US. We used the American Medical Association Graduate Medical Education Directory to identify them and obtain their contact information [14]. The study protocol was approved by the Institutional Review Board of the State University of New York (SUNY) at Buffalo.

### Survey questionnaire

The survey questionnaire consisted of 3 parts. The first part included questions about curricula for teaching the content of clinical practice guidelines [13]. The second part included questions about using educational games: support of their use in residency training (yes/no), current use (yes/no), type of educational games used (Jeopardy style, board game, other), and the purpose of using the games (for teaching, review, evaluation). The third part included questions about the characteristics of the residency program: geographical region (Northeast, South, Midwest, and West), type (community based, university based, military based), number of residents, and percentage of international medical graduates (<25%, 25-50%, 51-75%, >75%). It also included questions about the characteristics of the program director (sex, years as program director) (See Additional file 1).

The questionnaire was developed based on discussions with 5 chief residents and 2 program directors of internal medicine residency programs attending the 2005 annual meetings of the American College of Physicians (ACP) and the Association of Program Directors in internal medicine (APDIM). We pilot tested the questionnaire with 3 program directors (2 current, 1 former).

### Data collection

In April 2007, we invited the program directors by mail to participate in the survey. We attempted to maximize the response rate using the following strategies [15,16]: university sponsorship, personalized cover letter, colored ink, stamped return envelope, first class mailing, follow up mail (at 5 weeks), follow up fax (at 9 weeks), including a questionnaire in the follow up mail, and an interesting, short, and user friendly questionnaire with factual questions, and with more relevant questions first. We also included a Microsoft PowerPoint version of an educational game to teach clinical practice guidelines [8].

### Statistical analysis

First, we conducted descriptive analyses of the questions about the use of educational games. Next, we conducted exploratory regression analyses to explore the associations between residency program characteristics and the use of educational games (yes/no). We specifically used logistic models for each of the options for the questions about the use of educational games as the dependent variables. For each of these models, we used the specialty (family medicine vs. internal medicine), the program director characteristics, and the residency program characteristics as the independent variables. We chose the following as the respective reference categories for the categorical variables: internal medicine specialty, male sex, Northeast geographical region, community based programs, and <25% international medical graduates. We entered and managed data in Microsoft Office Access and conducted the analysis using SPSS 16.0 (SPSS, Inc., Chicago, Illinois).

## Results

**Figure 1:**
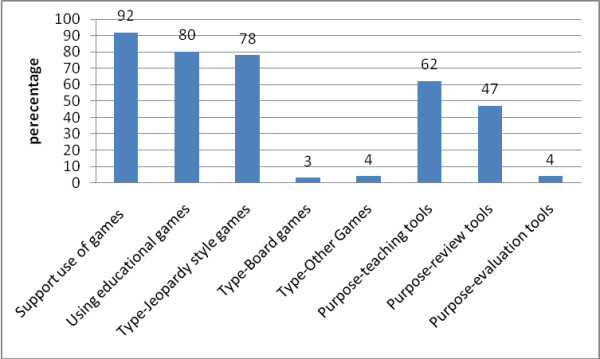
**Affirmative answers about use of educational games in residency program curricula (N = 434)**.

The overall response rate was 52% (434 out of 839) (35% after the initial mail and 44% after the follow up mail). Table 1 presents the characteristics of the program directors and of the residency programs. Table 2 and Figure 1 report the responding program directors' answers to questions about the use of educational games in their residency programs with the corresponding 95% confidence intervals.

**Table 1:** Characteristics of responding program directors and of their residency programs; (N = 434).

	n	% (95% CI)
Specialty (family medicine)	239	55 (52-58)

Sex of program director (female)	98	23 (20-26)

Geographical region		

Northeast	129	30 (27-30)

South	112	26 (23-29)

Midwest	119	27 (24-30)

West	68	16 (14-18)

Affiliation		

Community based	294	68 (65-71)

University based	113	26 (110-116)

Military based	15	3 (2-4)

Other	6	1 (0-2)

International graduates		

<25%	174	40 (37-43)

25-50%	85	20 (17-23)

51-75%	76	18 (15-21)

>75%	94	22 (17-23)

	**mean (SD)**	**mean (95% CI)**

Years as director, mean (SD)	7.3 (5.4)	7.3 (6.8-7.8)

Number of residents per program, mean (SD)	36.5 (28.5)	36.5 (33.8-39.2)

**Table 2:** Affirmative answers about use of educational games in residency program curricula (N = 434).

	n	% (95% CI)
Support use of games as educational strategy	401	92 (90-94)

Using educational games	347	80 (77-83)

Using Jeopardy style games	338	78 (75-81)

Using Board Games	13	3 (2-4)

Using Other Games	18	4 (5-6)

Using games as teaching tools	267	62 (59-65)

Using games as review tools	205	47 (44-50)

Using games as evaluation tools	17	4 (5-6)

### Support of the use of educational games

Ninety two percent of responding program directors were in support of the use of educational games as an educational strategy in residency training. Supporting the use of games was not associated with any of the investigated factors.

### Current use of educational games

Eighty percent of the responding program directors reported current use of educational games. Regression analyses revealed no significant associations except that programs with more than 75% international graduates were less likely to utilize educational games (OR = 0.33; 95% Confidence Interval (CI) 0.15-0.73).

### Type of games used

Seventy eight percent and 3% of the responding programs reported using Jeopardy like games and Board games, respectively. Using Jeopardy like games was inversely associated with >75% of residents in the program being international medical graduates (OR = 0.32; 95% CI 0.15-0.68). There were too few programs (3%) using board games to allow valid regression analyses.

### Purpose of using the games

The percentage of program directors who reported using educational games as teaching tools, review tools, and evaluation tools were 62%, 47%, and 4% respectively. Using educational games as teaching tools was not associated with any of the investigated factors. Using educational games as review tools was associated with a Southern geographic location of the program (OR = 2.01; 95% CI 1.11-3.63); inversely associated with family medicine specialty (OR = 0.44; 95% CI 0.25-0.76); inversely associated with university based program (OR = 0.52; 95% CI 0.30-0.90); and inversely associated with >75% of residents in the program being international medical graduates (OR = 0.50; 95% CI 0.26-0.90). There were too few programs (4%) using games as evaluation tools to allow valid regression analyses.

## Discussion

We conducted a survey of family medicine and internal medicine residency programs in the United States to explore their use of educational games. Support and use of educational games were high at 92% and 80%, with Jeopardy like games being the most frequently used (78%). While 62% and 47% of programs used games as teaching and review tools respectively, only 4% used them as evaluation tools.

This study has a number of strengths. To our knowledge, this is the first national survey exploring the use of educational games. The survey questionnaire was rigorously designed based on input from current and former program directors, and chief residents. The response rate (52%) was comparable to the mean response rate to surveys of physicians published in medical journals (54%) [16].

One limitation of this study is a possible selection bias if program directors not supporting or using educational games were less likely to respond. In that case, our findings would have overestimated the use of games. In the worst case scenario (i.e., assuming that all those who did not respond were not supporters or users), the percentage of program directors supporting or using educational games would be 48% and 41% respectively. Such percentages would still reflect relatively high support and use. Information bias is less likely here because of the factual nature of the questions, particularly the one about using educational games. The question about preference of using educational games is obviously more subjective and we did not test this question for reliability.

Educational games appear to be equally popular as educational tools among both family medicine and internal medicine residency programs. The reasons for the inverse association between using educational games and the percentage of international medical graduates in the program are not clear. These analyses were not defined a priori and should be interpreted with caution given the potential study limitations discussed above.

The strong support and high prevalence of use of educational games are intriguing in the face of the limited evidence supporting their effectiveness [4-6]. This remains concerning even when considering how the potential response bias could have overestimated the percentage of supporters and users of educational games. A possible explanation of these findings is that medical educators do not follow an evidence-based approach in selecting their educational strategies. For example, they might not search for the evidence relating to the educational interventions they employ. Also, they might give more value to perceived effectiveness - based on personal educational experience - compared to systematically evaluated effectiveness. This may particularly apply when the quality of studies providing the evidence is poor.

The lack of evidence for the effectiveness of educational games is not necessarily an evidence of the lack of their effectiveness. This lack of evidence might be related to an inadequate power of studies to show an effect and/or to the poor quality of those studies. Indeed, each of the three systematic reviews conducted in this area identified a limited number of studies that were of moderate methodological quality at best, and of poor reporting in general [4-6].

## Conclusion

### Implications for medical education practice

Given their potential effectiveness, medical educators might use educational games when other types of educational interventions (e.g., didactic lectures) are perceived or proven to have limited effectiveness. They would need to weigh possible but unproven benefits against the required investment (in money, time and effort) and against the opportunity cost of not using other potentially effective interventions [17]. This becomes particularly important when considering the limited time and financial resources available to residency programs.

### Implications for medical education research

Given their widespread acceptability and use, there is a need for more and better designed studies to assess the effectiveness of educational games. These studies should adhere to high methodological standards(such as allocation concealment and protection from contamination [18]), and evaluate relevant educational and clinical outcomes (e.g. behavioral change). A wider question to be explored is on how program directors and medical educators in general actually select certain educational strategies in the absence of best practice evidence.

## Competing interests

Two of the authors (EAA and HJS) are developing educational games. Otherwise, the authors report no academic, institutional, political, personal, financial or other conflicts of interest.

## Authors' contributions

Authors contributions: conception and design: EAA, RM, MCW, AM, HJS; acquisition of data: EAA, RM, MCW, AS, AM; analysis and interpretation of data: EAA, SG; drafting of manuscript: EAA, SG; critical revision of the manuscript for important intellectual content: EAA, SG, RM, MCW, AS, AM, HJS; obtaining of funding: EAA, RM, HJS. All the authors read and approved the final manuscript.

## Pre-publication history

The pre-publication history for this paper can be accessed here:

http://www.biomedcentral.com/1472-6920/10/26/prepub

## Supplementary Material

Additional file 1 **The survey questionnaire**. This additional file includes the questionnaire used in the survey study. Click here for file
