# Supplementary material for: Support for and aspects of use of educational games in family medicine and internal medicine residency programs in the US: a survey
Source: BMC Med Educ. 2010 Mar 25;10:26. doi: 10.1186/1472-6920-10-26 (PMC2851700; doi:10.1186/1472-6920-10-26)
Supplement: Additional file 1 — The survey questionnaire. This additional file includes the questionnaire used in the survey study. [file 1472-6920-10-26-S1.DOC]

***Educational games use the format of a game (e.g. Jeopardy, board games) in teaching***

1. I support using educational games as an educational strategy in residency training

 No  Yes

1. My program already uses educational games:

 No  Jeopardy style  Board game  Other: ________________

1. My program uses educational games as: (Check all that apply)

 Teaching tools  Review tools  Evaluation tools  N/A

_ _ _ _ _ _ _ _ _ _ _ _ _ _ _ _ _ _ _ _ _ _ _ _ _ _ _ _ _ _ _ _ _ _ _ _ _ _ _ _ _ _ _ _ _ _ _ _ _ _ _ _ _ _ _ _ _ _ _ _ _ _ _ _ _ _ _ _

1. US geographical region of residency program:

 Northeast  South  Midwest  West

1. Residency program best described as *primarily*:

 Community based  University based  Military based  Other:____

1. Total number of categorical Internal Medicine residents: ______
2. Percentage of residents who are international medical graduates:

 <25%  25-50%  51-75%  >75%

1. Program director:  Female  Male
2. Number of years as program director: ______
